# Speed, Change of Direction Speed and Reactive Agility in Adolescent Soccer Players: Age Related Differences

**DOI:** 10.3390/ijerph18115883

**Published:** 2021-05-30

**Authors:** Slobodan Andrašić, Marko Gušić, Mima Stanković, Draženka Mačak, Asim Bradić, Goran Sporiš, Nebojša Trajković

**Affiliations:** 1 Faculty of Economics, University of Novi Sad, 24000 Subotica, Serbia; andrasicslobodan@yahoo.com; 2 Faculty of Sport and Physical Education, University of Novi Sad, 21000 Novi Sad, Serbia; gusicmarko@yahoo.com (M.G.); macak.md@yahoo.com (D.M.); 3 Faculty of Sport and Physical Education, University of Niš, 18000 Niš, Serbia; mima.stankovic974@gmail.com; 4 Faculty of Kinesiology, University of Zagreb, 10000 Zagreb, Croatia; asim.bradic@kif.unizg.hr (A.B.); goran.sporis@kif.unizg.hr (G.S.)

**Keywords:** agility, differences, youth, performance, football

## Abstract

There are a plethora of studies investigating agility in soccer; however, studies have rarely presented the reaction time in differentiating age groups in adolescent soccer players. We investigated age differences in reactive agility, speed, and change of direction speed (CODs), in a group of highly trained adolescent soccer players. A total of 75 adolescent male soccer players (aged 14–19 years) were recruited. The players were grouped based on their age to under 15 (U15; *n* = 27), under 17 (U17; *n* = 25), and under 19 (U19; *n* = 23) players. Players were tested for 5 m, 10 m, and 20 m sprint, CODs speed test, Illinois test, and reactive agility test (total and reaction time). Only the reactive agility test with a live tester (RAT live) and RAT live reaction time (RAT live RT) distinguished U19 from both groups, U17 (RAT live, *p* < 0.01; RAT RT live, *p* < 0.01) and U15 (RAT live, *p* < 0.01; RAT RT live, *p* < 0.01). Groups did not have different times for 5 m sprint, RAT light and RAT RT light, F = 0.472, 2.691, 1.023, respectively, *p* > 0.05. Moreover, a significantly slower average performance of sprint 20, CODs left and right, and Illinois was also observed in U15 as compared to U17 and U19 (*p* < 0.05). We can conclude that results in agility tests that include live testers can be a significant factor that differentiates between adolescent soccer players considering their age.

## 1. Introduction

The demands of modern soccer have changed significantly and increased in recent years. Nowadays, players are required to have more power and to cover greater distances, with more frequent changes in intensity [1,2,3]. Most high-intensity activities (sprints) take place during decisive moments, such as tackling, offensive and defensive actions, as well as goal-scoring opportunities [4,5,6,7]. As soccer is considered to be a sport that requires that attackers evade their opponents’ pressures or tackles, and defenders reduce space on the field in order to limit attacking movements or potentially achieve a turnover, having good change of direction speed (CODs) and agility is beneficial [8]. In the last few decades, change of direction speed and reactive agility were considered to be the same skill [9]. However, nowadays, pre-planned agility may be defined as sprints with change of direction, while the reactive agility (RA) is classified as sprints with directional changes in response to a stimulus [10,11]. Therefore, RA is based on greater levels of motor control, when compared to pre-planned CODs [12].

Reactive agility and CODs are one of the most important skills required for soccer success [13]. Moreover, reactive agility tests (RATs) are able to differentiate the key performance indicators presented as the skill levels among soccer players [14]. On the other side, there are still some doubts about the test design and type of stimulus presented in the existing literature regarding reactive agility tests. In addition, most of the studies only measured total times instead of reaction times. As RATs are designed to evaluate both physical, and technical and cognitive abilities, there is an absolute need to further examine reactive agility assessment in soccer [15].

Some research shows that older adolescent players tend to complete the RATs quicker than younger players due to their higher fitness level, CODs, and anticipatory skill, which makes them play on a much higher level [13]. Several studies investigated the perceptual abilities of higher-level players, and it was shown that there are fundamental perceptual and cognitive differences between them and lower-level players [16,17,18]. Additionally, Tretloci et al. [19] showed that field-based tests including vertical jumps, change of direction speed, and reactive agility can differentiate between under 16 elite and sub-elite soccer players. This was confirmed by Trajkovic et al. [20], showing that the skilled players performed better in reactive agility tests, speed, and CODs compared to amateur players. Moreover, the authors stated that reactive agility tests with live opponent stimuli can be a significant factor that differentiates between adolescent soccer players considering their level.

At this point, there are studies that determine the difference in physical performance between levels of play. However, there are not many studies that show the difference between age groups in CODs and RA [13,21]. Additionally, the categories used in those studies do not match the categories used here.

Moreover, according to the authors’ knowledge, there are no studies that compare light and live stimulus in RATs in soccer players to assess whether these approaches differentiate between different age groups. Therefore, the purpose of this study was to determine possible age-related differences in speed, CODs and reactive agility in a group of trained adolescent soccer players.

## 2. Materials and Methods

### 2.1. Subjects

A priori, the G*power 3.1 power analysis software was used to determine that the required sample size is *n* = 72 given the critical F(69) = 3.13, eta2 = 0.13 *p* = 0.05, 1 − β = 0.8, and number of groups = 3. A total of 75 adolescent male soccer players (aged 14–19 years), who participate at the highest level of competition in Serbia at their age, were recruited for this research (Table 1). The players were grouped based on their age to under 15 (U15; *n* = 27), under 17 (U17; *n* = 25), and under 19 (U19; *n* = 23) players. Only field players were tested, with goalkeepers excluded. Written informed consent was obtained from the players and their parents. Moreover, the ethics board of the Faculty of Sport and Physical Education provided the approval of the research experiment (Ethical Board Approval No: 2019/31). Players were recruited if they had at least 5 years of experience in playing soccer; had a general training history (more than three times per week) in the previous 12 months; were currently training for soccer (more than 7 h per week); and did not have existing medical conditions that would compromise study participation.

### 2.2. Procedure

Testing was conducted at the beginning of the annual training season to limit differences in training status between players. All players followed a similar training program under the supervision of their respective coaches. All performance tests were conducted on the same day. Test sessions were undertaken between 09:00 and 13:00 h following at least 8 h of sleep and 48 h of rest. All performance tests were performed on an outdoor facility with artificial grass in favorable weather conditions (no wind or rain). Before testing, a 20 min standardized warm-up was conducted, which consisted of low-intensity running, acceleration runs, skipping and hopping exercises. Players were familiar with all test procedures.

*Height and weight* measurements were taken in the morning. Height was measured with a fixed stadiometer (+0.1 cm, Holtain Ltd., Crosswell, UK), and body mass with a digital balance (+0.1 kg, ADE Electronic Column Scales, Hamburg, Germany). The same researcher conducted all the measurements.

*Running speed*. The running speed of players was determined using the time to 5, 10 and 20 m using infrared timing gates, 20-m sprint effort with photocell gates (Microgate, Polifemo Radio Light, Bolzano, Italy) placed 0.4 m above the ground, with an accuracy of 0.001 s. The timer was automatically activated as participants crossed the first gate at the starting line with split times at 5 m and 10 m. Players were instructed to run as quickly as possible over the 20-m distance from a standing start (crouched start positioned 0.5 m behind the timing lights). Acceleration was evaluated using the time to cover the first 5 m of the 20-m test. Participants performed two trials with at least 3 min of rest between them. The best performance of the two tests was used for analysis.

*Change of direction speed test (CODs)*. The pre-planned agility test [22] is used to evaluate CODs. Participants were asked to sprint as fast as possible for 5 m through a triggered timing gate (start gate), make a 45° cut and sprint 5 m to the left and right through a target gate. In this test, participants knew the cut direction. Running time was recorded using photocell gates (Microgate, Polifemo Radio Light, Bolzano, Italy) placed 0.4 m above the ground, with an accuracy of 0.001 s at the start and finish gates. The best time of three attempts on the left and right side was considered for further analysis.

*The reactive agility test (RAT)* was performed according to the protocol described previously by Chaouachi et al. [23]. In the current study, the RAT involved a decision-making element provided by a live tester (RAT live) acting as an opponent and light stimuli used instead of testers (RAT light). During RAT live, the tester had 4 options for each condition: preplanned and randomly ordered (i.e., 8 trials). All these conditions were provided to each player in 2 series (5–8 min between sets rest) in a random order. Players were instructed to recognize the cues as soon as possible. Running time was recorded using photocell gates (Microgate, Polifemo Radio Light, Bolzano, Italy) placed 0.4 m above the ground, with an accuracy of 0.001 s. Total time (RAT TT live) and reaction time (RAT RT live) were recorded for each trial, and the best performance was considered for the analysis. The same conditions were used for RAT light, but this time the Witty SEM lights were used instead the testers. When the participants pass the first gate, the signal shows right or left direction. The participants must react to the visual signal, change direction and pass the third gate. Similar to RAT live, the total time (RAT TT light) and response movement time (RAT RT light) were recorded for each trial, and the best performance was taken for analysis.

*Illinois agility test*: The length of the field is 10 m, while the width (distance between the start and finish points) is 5 m. Four cones were placed in the center of the testing area at a distance of 3.3 m from one another. Four cones were used to mark the start, finish and two turning points. The subjects started the test lying face down, with their hands at shoulder level. The trial started on the “go” command, and the subjects began to run as fast as possible. The trial was completed when the players crossed the finish line without having knocked any cones over. Best time out of three trials was used for analysis [24].

### 2.3. Statistical Analysis

The analysis of the data obtained from the study was performed by SPSS software, version 23.0 (SPSS Inc., Chicago, IL, USA). Data are reported as mean ± SD unless otherwise stated. The Kolmogorov–Smirnov test was conducted to verify if all data met the normality test assumption. Test–retest reliability was assessed for all tests using a one-way intra-class correlation coefficient (ICC) based on average measurements (ICC 1,k).

All analyses of variance (ANOVA) were performed on log-transformed data; for the sake of clarity, however, they are reported non-transformed. Age-based comparisons of study outcomes were made with one-way ANOVA (U15, U17, and U19). When ANOVA showed a significant group effect, between group differences were allocated by using post hoc Bonferroni tests. Eta squared (ŋ^2^) is reported as a measure of effect size and defined as small (0.01), medium (0.06), and large (0.14) according to Cohen. The level of significance was set at *p* < 0.05.

## 3. Results

### 3.1. Sample Characteristics

Table 1 shows the physical characteristics of the players according to age group. Height and weight were similar across the groups (*p* > 0.05); therefore, adjustments were not performed.

### 3.2. Study Outcomes in Relation to the Age Group of Soccer Players

On average, the U15 needed significantly more time than U19 to perform all tests, except sprint 5 m (*p* = 0.63), RAT light (*p* = 0.08), and RAT RT light (*p* = 0.37), which were similar across the age groups. A significantly slower average performance of sprint 20, CODs left and right, and Illinois was also observed in U15 as compared to U17 and U19. However, the U17 had a significantly slower mean performance only of RAT live and RAT RT live than the U19. Visit Table 2 for detailed results from a one-way analysis of variance.

## 4. Discussion

The present study aimed to determine the difference in several performance indicators relevant for soccer performance in adolescent players of different age groups. The main finding of this study was that the reactive agility test with live testers was able to differentiate U19 players from other age groups. Moreover, the U15 group showed slower average performance in sprint 20, CODs left and right, and Illinois compared to U17 and U19. A possible explanation for these results could be found in the fact that modern soccer training, with frequent changes in tactics based on the characteristics of the opponent, has led to increased adaptability of player roles, especially in young players [9]. Moreover, it is not so uncommon in youth training to change a player’s position in response to different game situations, developing a large range of technical solutions useful for their future soccer-playing career [25].

In the literature, sprinting ability over short (5 m) and longer distances (20 m) is considered to require separate and specific biomechanical and neuromuscular qualities and, therefore, training techniques [26]. When considering the U15, U17, and U19 players, we found a difference between groups for 10 m and 20 m distance, which is in line with the abovementioned fact. The current findings [14] show that there is no difference between U17 and U19 for 20 m sprints which is, as well, presented in this study. In addition, we found that U15 players had worse results than U17 and U19 players. Generally, most studies that investigated age difference came to the conclusion that older age groups had better results than younger groups [14,27]. These differences could be associated with the maturity stage of players, which can affect U15 sprint performance more [28] compared to U17 and U19. Moreover, post-pubertal players have accelerated gains in strength supported by rapid gains in muscle mass [29], which may also contribute to the mentioned differences.

Our findings suggest that U19 and U17 players, who performed with similar results, were statistically significantly better at CODs left, CODs right and the Illinois test than U15 players. These results are similar to the ones found by [9], where U16 and U18 players gave better results in the Illinois modified test. Additionally, their study showed that there was no difference in the CODs left and CODs right test between U16 and U18 players, but there were differences found between U14 players and the aforementioned groups. A possible reason for non-existent differences between U19 and U17 players is that the greatest individual differences in biological maturation were found in players 11–16 years old [30]. Contrary to this study [14], Poljskic et al. concluded that U19 players were significantly better that U17 players. Taking everything into account, the superiority of U19 players in agility performances may be observed as a direct consequence of their long involvement in systematic soccer training and higher performance level due to the CODs.

Our results show that U19 players scored better in the RAT live test than U15 and U17 players. Additionally, we found that U19 players had better results than U15 and U17 in reaction time with live stimuli. Therefore, in the current study, RATs clearly discriminated U19 players from U15 and U17. A possible reason could be found in better anticipatory skills in older adolescent players who have more games and experience behind them. They have better ability to recognize relevant cues of testers, as previously demonstrated for team sport athletes [31,32]. Our results are similar to Fiorilli et al. [13], where U16 and U18 players made better results than U14 in the reactive agility test. Moreover, Poljskic et al. [14] showed that U19 players had better results than U17 in RAG (reactive agility) test. On the other hand, there is a study that found no significant difference between juniors (<18) and seniors (>18) in specific reactive agility tests [33]. However, reactive agility is being developed until the late adolescent age, when it can reach its peak, which could be the reason for the discrepancy in the results.

It has been stated recently that intervention programs may have to be different for different age stages [34]. According to our results, we could speculate that modern training is similar for all age categories in adolescent soccer players. Nowadays, the training contains sport-specific stimuli rather than generic and high-intensity training for physical skills. Soccer players, who have anticipatory expertise and make decisions much faster, are able to recognize and react promptly to a stimulus. Younger players with less experience may need more time to respond to a stimulus before having the proper reaction in the shortest time possible in order to avoid being executed by the opponent.

The main limitation of this study is that the attribution of physical ability could be to talent or previous training. In our study, the players were interviewed about their current training load (weekly time) and previous experience (years engaged in soccer). Moreover, they were from the same squad, with the same programs conducted in all categories. Therefore, we could speculate if different approaches to training could contribute to differences in other variables. Future studies should examine players from different teams and academies. Moreover, the maturity level was not introduced and taken into account due to the fact that the majority of studies have focused largely on players 11–16 years of age, where individual differences in biological maturation are perhaps the greatest. Another limitation is the possibility of the circadian rhythm’s influence on performance [35] due to the time the testing was conducted (9:00 to 13:00 h).

## 5. Conclusions

Reactive agility and COD speed are key skills required for soccer success, based on greater levels of motor control. We found that the reactive agility test with a live tester can be a significant factor that differentiates between older and younger adolescent soccer players. Moreover, our findings prove that field-based tests including speed, change of direction speed, and reactive agility are sufficiently sensitive to differentiate between a group of adolescent soccer players. Further studies are needed to confirm these results.

## Figures and Tables

**Table 1:** Physical characteristics for U15 (*n* = 25), U17 (*n* = 27), and U19 (*n* = 23).

	U15	U17	U19
Age	14.7 ± 0.6	16.2 ± 0.7	18.8 ± 0.7
Height	178.06 ± 5.82	180.49 ± 6.56	179.12 ± 5.45
Weight	69.06 ± 10.82	72.89 ± 8.48	70.70 ± 8.22
Experience (years)	5.1 ± 2.7	6.5 ± 2.9	7.3 ± 1.4
Training (min∙week-1)	447 ± 126	475 ± 167	487 ± 130

U15—younger than 15 years; U17—younger than 17 years; U19—younger than 19 years; min∙week—minutes per week.

**Table 2:** Times for 5 m, 10 m sprint, and 20 m sprint, and agility performance for the U15 (*n* = 25), U17 (*n* = 27), and U19 (*n* = 23) soccer players.

Outcomes	U15	U17	U19	A One-Way ANOVA	
				F_(1, 144)_	ŋ^2^
Sprint 5 m	1.16 ± 0.23	1.13 ± 0.19	1.11 ± 0.12	0.47	0.02
Sprint 10 m	1.93 ± 0.13 ^a^	1.85 ± 0.18	1.83 ± 0.11	3.28 *	0.09
Sprint 20 m	3.38 ± 0.23 ^b^	3.18 ± 0.27	3.16 ± 0.31	5.17 **	0.16
CODs left	2.25 ± 0.15 ^b^	2.15 ± 0.17	2.12 ± 0.21	3.61 *	0.07
CODs right	2.27 ± 0.14 ^b^	2.16 ± 0.19	2.13 ± 0.16	4.87 **	0.11
Illinois	15.82 ± 0.76 ^b^	15.24 ± 0.53	14.93 ± 0.58	12.47 **	0.25
RAT light	2.69 ± 0.13	2.61 ± 0.16	2.60 ± 0.19	2.69	0.07
RAT live	2.58 ± 0.10	2.58 ± 0.11	2.48 ± 0.06 ^c^	8.99 **	0.20
RAT RT light	1.44 ± 0.16	1.41 ± 0.16	1.38 ± 0.11	1.02	0.03
RAT RT live	1.49 ± 0.08	1.38 ± 0.10	1.31 ± 0.14 ^c^	16.27 **	0.31

Values are mean ± SD. U15—younger than 15 years; U17—younger than 17 years; U19—younger than 19 years; CODs left—change of direction left; CODs right—change of direction right; RAT light—reactive agility test with witty SEM visual signals; RAT RT light—reaction time during RAT live; RAT live—reactive agility test with testers; RAT RT light—reaction time during RAT light; * significant age group effect at *p* ≤ 0.05; ** significant age group effect at *p* ≤ 0.01; ^a^ U15 and U19 significantly different at *p* ≤ 0.05; ^b^ U15 significantly different at *p* ≤ 0.05; ^c^ U19 significantly different at *p* ≤ 0.05.

## Data Availability

The data presented in this study are available on request from the corresponding author. The data are not publicly available because they contain information that could compromise the privacy of research participants.
